# Neurogranin as a cognitive biomarker in cerebrospinal fluid and blood exosomes for Alzheimer’s disease and mild cognitive impairment

**DOI:** 10.1038/s41398-020-0801-2

**Published:** 2020-04-29

**Authors:** Weilin Liu, Huawei Lin, Xiaojun He, Lewen Chen, Yaling Dai, Weiwei Jia, Xiehua Xue, Jing Tao, Lidian Chen

**Affiliations:** 1 College of Rehabilitation Medicine, Fujian University of Traditional Chinese Medicine, Fuzhou, Fujian China; 2 Department of Biochemistry and Molecular Biology, University of Oklahoma Health Sciences Center, Oklahoma City, OK USA; 3 The Academy of Rehabilitation Industry, Fujian University of Traditional Chinese Medicine, Fuzhou, Fujian China; 4 Affiliated Rehabilitation Hospital, Fujian University of Traditional Chinese Medicine, Fuzhou, Fujian China

**Keywords:** Prognostic markers, Learning and memory

## Abstract

Alzheimer’s disease (AD) is a progressive neurodegenerative disorder with clinical, biological, and pathological features occurring along a continuum from normal to end-stage disease. Currently, the diagnosis of AD depends on clinical assessments and post-mortem neuropathology, which is unbenefited early diagnosis and progressive monitoring. In recent years, clinical studies have reported that the level of cerebrospinal fluid (CSF) and blood neurogranin (Ng) are closely related to the occurrence and subsequent progression of AD. Therefore, the study used meta-analysis to identify the CSF and blood Ng levels for the development of diagnosis biomarker of patients with AD and mild cognitive impairment (MCI). We searched the Pubmed, Embase, Cochrane Library, and Web of Science databases. A total of 24 articles eligible for inclusion and exclusion criteria were assessed, including 4661 individuals, consisting of 1518 AD patients, 1501 MCI patients, and 1642 healthy control subjects. The level of CSF Ng significantly increased in patients with AD and MCI compared with healthy control subjects (SMD: 0.84 [95% CI: 0.70–0.98], *P* < 0.001; SMD: 0.53 [95% CI: 0.40–0.66], *P* = 0.008), and higher in AD patients than in MCI patients (SMD: 0.18 [95% CI: 0.07–0.30], *P* = 0.002), and CSF Ng level of patients with MCI-AD who progressed from MCI to AD was significantly higher than that of patients with stable MCI (sMCI) (SMD: 0.71 [95% CI: 0.25–1.16], *P* = 0.002). Moreover, the concentration of Ng in blood plasma exosomes of patients with AD and MCI was lower than that of healthy control subjects (SMD: −6.657 [95% CI: −10.558 to −2.755], *P* = 0.001; and SMD: −3.64 [95% CI: −6.50 to −0.78], *P* = 0.013), and which in patients with AD and MCI-AD were also lower than those in patients with sMCI (*P* < 0.001). Furthermore, regression analysis showed a negative relationship between MMSE scores and CSF Ng levels in MCI patients (slope = −0.249 [95% CI: −0.003 to −0.495], *P* = 0.047). Therefore, the Ng levels increased in CSF, but decreased in blood plasma exosomes of patients with AD and MCI-AD, and highly associated with cognitive declines. These findings provide the clinical evidence that CSF and blood exosomes Ng can be used as a cognitive biomarker for AD and MCI-AD, and further studies are needed to define the specific range of Ng values for diagnosis at the different stages of AD.

## Introduction

Alzheimer’s disease (AD) is a neurodegenerative disease with insidious and progressive onset of disease. It is also one of the most common types of dementia and an important cause of death in the elderly, with more than 60% of all the dementia cases among elderly over 65 years old attributed to AD^1^. Currently, ~35 million people are suffering from AD or other types of dementia, and the number of dementia patients worldwide is predicted to reach 80 million by 2050, with bringing enormous economic pressure to both the family and society^2^. As AD is clinically found to be relatively advanced, a large number of literatures have shown that the treatment of AD is poor ^3,4^.

AD is characterized by progressive loss of memory and impairment of cognitive ability, but before showing typical symptoms of dementia, patients will have a pre-clinical mild cognitive impairment stage^5^. Mild cognitive impairment (MCI) is a condition in which an individual exhibits a slight decline in cognitive ability that is not perceived, and the early stage of AD does not impair the patient’s ability of living and working, but if it is not prevented from developing in time, more than 50% of people with MCI will likely progress to dementia^6^. Therefore, there is an urgent need for cost-effective biomarkers for early diagnosis of AD.

Although the hallmark pathological features of AD, amyloid-β (Aβ) deposition, phosphorylated Tau protein to form neurofibrillary tangles, and sustained neuroinflammatory reactions, have long been described in the brain^7–9^. These important pathological changes lead to dysfunction and loss of neurons and synapses. Unfortunately, many intervention therapies targeting Aβ and Tau have been unsuccessful^10^. Thus, we need further investigation on the etiology of AD and subsequent therapeutic intervention. In the past few years, accumulated evidence suggests that neurogranin (Ng) as a post-synaptic protein is closely related to synaptic loss in AD patients^11^.

Ng is a neuron-specific and post-synaptic protein that binds to calmodulin and is abundantly expressed in the brain, particularly in the dendritic spine of hippocampus and cerebral cortex^12^. It is thought to be involved in synaptic plasticity and long-term potentiation (LTP) by regulating calcium-mediated signaling pathways^13^. There are many studies demonstrating that in animals, Ng knockdown models inhibit synaptic LTP and impact cognitive function^13^, whereas upregulation promotes LTP and improves cognition^14^. Similar findings also have been found that Ng is depleted in the brain, rose in cerebrospinal fluid (CSF), and associated with poorer cognitive performance in AD patients^15^. Ng has abundant neuronal expression in the central nervous system and compared with traditional biomarkers, Ng can detect the pathological changes of AD at an earlier stage, even in the MCI stage, so far it is considered to be a potential biomarker for diagnosis of AD^16^. This study aimed to investigate Ng whether as a cognitive biomarker in the CSF and blood for diagnosis of AD and MCI based on high-level medical evidences. Therefore, we measured CSF and peripheral blood Ng levels in three groups of populations, healthy subjects, MCI patients, and AD patients, from cross-sectional and longitudinal studies.

## Methods

The meta-analysis is conducted in strict accordance with the requirements of the Preferred Reporting Items for Systematic Reviews and Meta-analysis (PRISMA) statement^17^ and was registered at International Prospective Register of Systematic Reviews (https://www.crd. york.ac.uk/PROSPERO/) (numbers CRD42019135344 and CRD42019141393).

### Search strategy

Two individuals independently searched all English articles using the Pubmed, Embase, Cochrane Library, and Web of Science databases through June 1, 2019. The search term was (Neurogranin OR NRGN OR Hng OR Protein Kinase C Substrate OR RC3) AND (Alzheimer’s disease OR Mild cognitive impairment). The initial search generated 194 records from Pubmed, 708 records from Embase, and 17 records from the Cochrane Library, 527 records from the Web of Science database.

### Inclusion and exclusion criteria

#### Inclusion criteria

The articles need to meet the following characteristics: (1) they must meet the diagnosis criteria of AD and MCI according with NINCDS-ADRDA Criteria^18^, or NIA-AA Criteria^19,20^, or IWG-2 Criteria^21^. (2) The Ng concentration of CSF or blood must be one of the main variables of interest in patients with AD or MCI. (3) The reported data on Ng in at least two groups of subjects (AD, MCI, and healthy control subjects).

#### Exclusion criteria

First, we excluded the articles that were unrelated to Ng, MCI, or AD, or if they were review articles or animal experiments. In addition, studies were excluded that only reported the levels of Ng for participants with MCI or AD without control group. Thirdly, if articles did not have necessary Ng data, we also excluded them. In the end, we included 24 studies that fulfilled both the inclusion and exclusion criteria (Supplementary Fig. 1).

### Data extraction

Two different individuals extracted the following data for the purposes of this meta-analysis. We extracted sample sizes, mean Ng concentrations or median, standard deviations or interquartile range, and we regarded them as the primary outcome. In addition, data on the author and publication year of the article, the average age of the patient, the gender distribution, the Mini-Mental State Examination (MMSE) score, the sample source (CSF or blood or serum or plasma or exosomes), and the assay type were also extracted.

### Quality assessment

We used a checklist of Cross-sectional Study/Prevalence Quality to assess the methodological quality of the studies which was recommended by Agency for Healthcare Research and Quality (AHRQ), and AHRQ checklist consists of 11 items^22^ (https://www.ncbi.nlm.nih.gov/books/NBK35156/). The 11-item ranges from 0 to 8 stars for cross-sectional studies. If an item was answered ‘NO’ or ‘UNCLEAR’, it would be scored ‘0’, if it was answered ‘YES’, then the item scored ‘1’. Article quality was evaluated as follows: low quality = 0–3; moderate quality = 4–7; high quality = 8–11.

In addition to the longitudinal observational study, we chose the Newcastle-Ottawa Scale (NOS) that is recommended by the Cochrane Collaboration^23^. The NOS ranges from 0 to 9 stars for case–control studies and cohort studies. A study can be awarded a maximum score of 1 star for each numbered item within the Selection and Exposure categories. A maximum of 2 stars can be given for comparability. A score above 5 implies that the study has high quality.

### Statistical analysis

We used the Stata 14.0 (Stata Corp) software to perform all statistical analyses. For certain studies with only median and interquartile ranges available in the articles, we need to estimates the means and standard deviations according to Hozo^24^ and Dehui Luo^25^. Then, we calculated standardized mean difference (SMD) and 95% confidence intervals and generated a forest plots to compare the mean Ng levels between AD or MCI and healthy control in order to eliminate the effects between different measurement scales. The random-effects model was chosen because we assumed heterogeneity between the articles. In the next statistical analysis, first, we performed overall meta-analysis between AD vs. healthy control, MCI vs. healthy control, and AD vs. MCI. Whether there is heterogeneity between the articles was evaluated by *Q* test, and the statistical significance was set to *P*-value < 0.1. We further used the *I*^2^ index to evaluate the influence of heterogeneity, *I*^2^ was 0.25, 0.50, and 0.75, which suggested small, moderate and high levels of heterogeneity^26^, respectively. Secondly, we conducted a subgroup meta-analysis study to test whether there was in significant difference in age-matched or mismatched groups, analytical method for Ng, different MMSE groups (≥20 and <20) and type of study. Thirdly, we performed a series of meta-regression analyses to assess the effect of multiple regulatory variables on SMD. By estimating the impact of a single study on the results of the meta-analysis, sensitivity analysis was also performed to test the robustness of the results. Then, we generated a funnel plot and chosen Egger test to evaluate the potential for publication bias for each meta-analysis. For publication bias, we adopted the trim and fill method to supplement the missing articles and verify if the results were robust by comparing them with the results before supplementing. All statistical significances were set at *P*-value < 0.05 except where noted, *P*-value below 0.1 was reported as a trend.

## Results

### Search results

According to our search strategy, we obtained a total of 1446 articles from four databases. Among them, we removed the duplicated 292 articles. By screening the titles and abstracts, we removed 1119 articles that were not related to the topic or reviews and animal experiments, and then according to our inclusion and exclusion criteria, we removed 11 more articles for the following reasons: lack of necessary Ng data, articles without healthy controls and date on brain of tissues instead of CSF, and finally, we received 24 articles that met the inclusion criteria^27–50^. The Flow Diagram showed the detailed process of selection (Supplementary Fig. S1). The characteristics of the 24 included studies are shown, including 4661 individuals, consisting of 1518 AD patients, 1501 MCI patients, and 1642 healthy control subjects (see Supplementary Table S1 for details).

### Quality assessment

A total of 17 cross-sectional and 7 longitudinal studies were included in this review. We evaluated the quality of these articles according to the corresponding scales (Supplementary Tables S2 and S3). The results show that the scores of articles assessed by 11-item ranged from 4 to 8 (mean = 6.2) and all of articles are in the moderate quality range, while the studies evaluated by the NOS ranged from 3 to 7 (mean = 5.7) and the overall quality of the literature is higher.

### Association of cerebrospinal fluid Ng with cognitive impairment in patients with AD and MCI

**Fig. 1:**
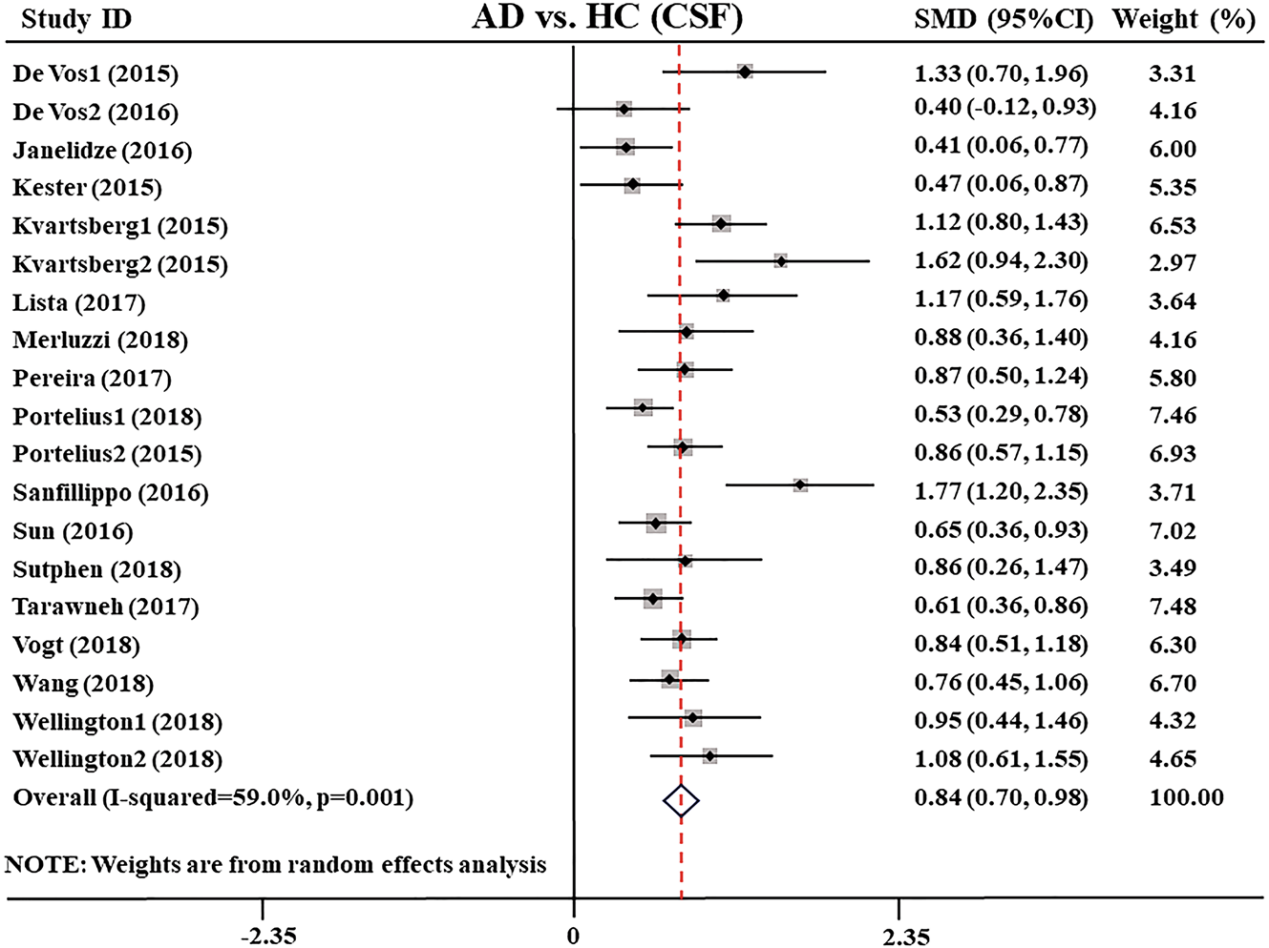
Forest plot of random-effects meta-analysis of cerebrospinal fluid Ng levels between AD patients and HC subjects. Data include 2891 individuals from 19 studies. The squares indicate individual study SMD and their corresponding 95% CIs and the sizes of the squares are proportional to study weight. Ng neurogranin, SMD standard mean difference, CI confidence interval, AD Alzheimer’s disease, HC healthy control.

First, we compared CSF Ng levels between AD patients and HC subjects, extracting data from 2891 individuals in 19 studies^27,28,31,32,34–48^. Random-effects meta-analysis was performed, and the results showed that CSF Ng levels were significantly elevated in AD patients compared with healthy control subjects (SMD: 0.84 [95% CI: 0.70–0.98], *z* = 11.72, *P* < 0.001, Fig. 1 and Supplementary Table S4). However, high heterogeneity between studies was observed (*Q* = 36.19, df. =18, *I*^2^ = 59.0, *P* = 0.001, Supplementary Table S4), and sensitivity analysis indicated that our results were not over-affected by a particular study.

**Fig. 2:**
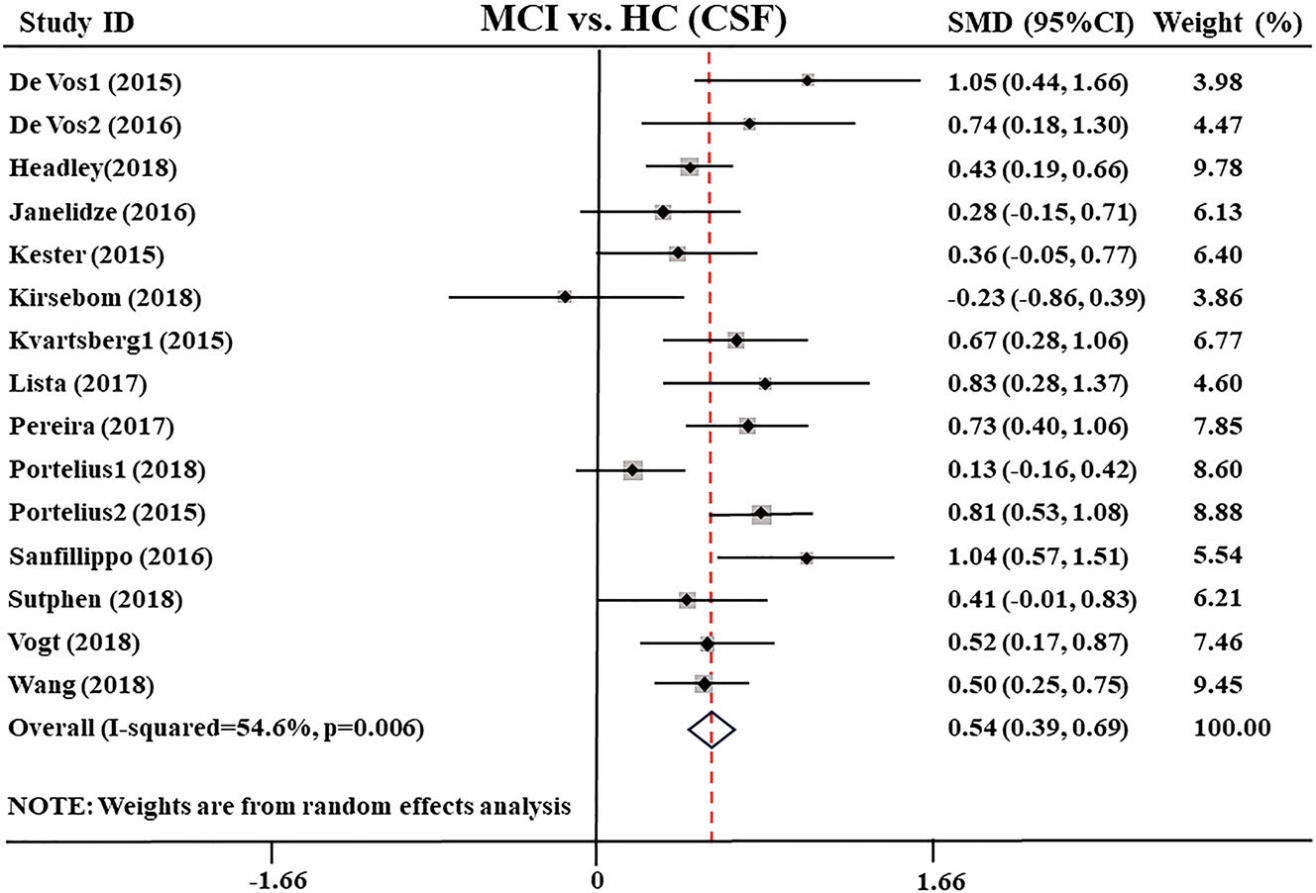
Forest plot of random-effects meta-analysis of cerebrospinal fluid Ng levels between MCI patients and HC subjects. Data include 2203 individuals from 15 studies. The squares indicate individual study SMD and their corresponding 95% CIs and the sizes of the squares are proportional to study weight. Ng neurogranin, SMD standard mean difference, CI confidence interval, MCI mild cognitive impairment, HC healthy control.

Then, we compared the CSF Ng levels between MCI and healthy control, including data of 2203 individuals from 15 studies^27,28,30–34,36,38–41,43,45,46^. The results showed that CSF Ng levels were significantly higher in MCI patients compared with healthy control subjects (SMD: 0.5 [95% CI: 0.39–0.6], z = 7.29, *P* < 0.001, Fig. 2 and Supplementary Table S4). There was moderate heterogeneity between studies (*Q* = 30.83, df. =14, *I*^2^ = 54.6, *P* = 0.0064, Supplementary Table S4), and sensitivity analysis showed the conclusions were not affected.

**Fig. 3:**
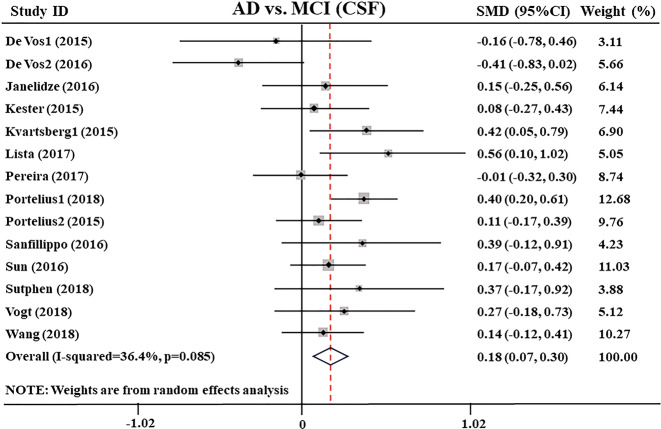
Forest plot of random-effects meta-analysis of cerebrospinal fluid Ng levels between AD and MCI patients. Data include 2216 individuals from 14 studies. The squares indicate individual study SMD and their corresponding 95% CIs and the sizes of the squares are proportional to study weight. Ng neurogranin, SMD standard mean difference, CI confidence interval, AD Alzheimer’s disease, MCI mild cognitive impairment.

Moreover, the distinctions between AD and MCI in CSF Ng levels, extracted from 14 studies^27,28,31,32,34,36,38–43,45,46^ encompassing a sample of 2216 individuals, were also tested. The comparison showed that the CSF Ng level in the AD group was higher than that in the MCI group (SMD: 0.18 [95% CI: 0.07–0.30], *z* = 3.05, *P* = 0.002, Fig. 3 and Supplementary Table S4). The heterogeneity between the studies was low (*Q* = 20.44, df. =13, *I*^2^ = 36.4, *P* = 0.085, Supplementary Table S4) and was not significantly affected by the specific study.

**Fig. 4:**
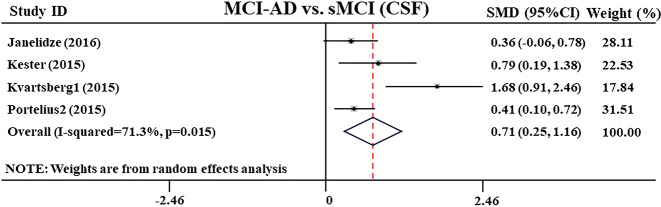
Forest plot of random-effects meta-analysis of CSF neurogranin levels between MCI-AD and sMCI patients. Data include 360 individuals from four studies. The squares indicate individual study SMD and their corresponding 95% CIs and the sizes of the squares are proportional to study weight. MCI-AD mild cognitive impairment patients who progressed to Alzheimer’s disease, sMCI stable mild cognitive impairment, CSF cerebrospinal fluid, SMD standard mean difference, CI confidence interval.

In addition, from four studies^31,32,34,40^, the researchers conducted follow-up of MCI patients for different lengths of time, and measured the CSF Ng level of patients with MCI-AD who progressed from MCI to AD. The results showed that the CSF Ng level of patients with MCI-AD was significantly higher than that of patients with stable MCI (sMCI) (SMD: 0.71 [95% CI: 0.25–1.16], *z* = 3.06, *P* = 0.002, Fig. 4 and Supplementary Table S4).

Furthermore, meta-regression analysis result showed a negative relationship (slope = −0.249 [95% CI: −0.003 to −0.495], *P* = 0.047, Supplementary Fig. S2 and Supplementary Table S5) between the MMSE scores and effect size in MCI patients, indicating that the lower the MMSE scores, the SMD increased implying larger CSF Ng levels compared with healthy control subjects.

### Association of blood Ng with cognitive impairment in patients with AD and MCI

We found that blood plasma Ng levels were determined for AD in two studies^27,35^. Our meta-analysis results showed that there was no statistical difference in plasma Ng levels between AD patients with healthy control subjects (SMD: −0.252 [95% CI: −0.663 to 0.1590], *z* = 1.20, *P* = 0.230, Supplementary Fig. S3), which was consistent with their respective findings.

**Fig. 5:**
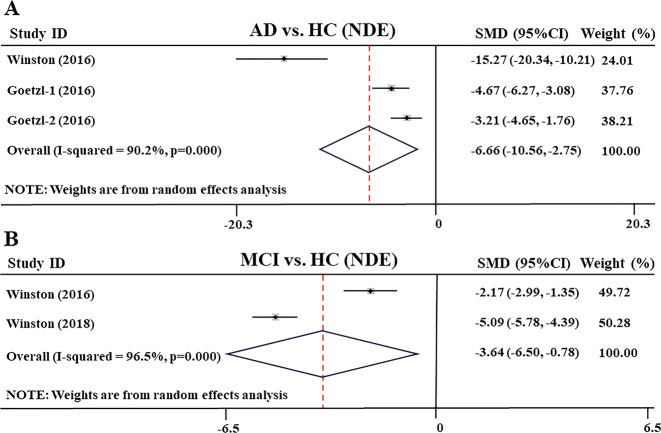
Forest plot of random-effects meta-analysis of blood plasma neuronal derived exosomes Ng levels between AD, MCI patients and HC subjects. **a** Data include 62 individuals from two studies (Goetzl’s study reports results from two groups of AD patients and HC subjects) for meta-analysis of exosomes Ng levels between AD and HC. **b** Data include 187 individuals from two studies for meta-analysis of exosomes Ng levels between MCI and HC. The squares indicate individual study SMD and their corresponding 95% CIs and the sizes of the squares are proportional to study weight. SMD standard mean difference, CI confidence interval, AD Alzheimer’s disease, MCI mild cognitive impairment, HC healthy control.

However, in blood exosomes, meta-analysis results from two other studies^29,49^ showed that compared with the healthy control subjects, the levels of blood plasma neuronally derived exosome (NDE) Ng in AD patients have an obviously decrease (SMD: −6.657 [95% CI: −10.558 to −2.755], *z* = 3.34, *P* = 0.001, Fig. 5a). In addition, Abner et al.^51^ reported that the concentration of blood plasma NDE Ng was reduced in older cognitively intact subjects tested by two samples collected at 3- and 11-year intervals (285.1 ± 76.8 pg/mL vs. 224.8 ± 61.0 pg/mL, *P* < 0.05), especially those subjects who transitioned to AD that the level of NDE Ng decreased significantly. These results were similar to the decreased Ng levels seen in human brain tissue of AD patients^52^.

Moreover, from two studies^49,50^, in blood exosomes, meta-analysis showed that the levels of blood plasma NDE Ng were significantly lower in MCI patients than in healthy control subjects (SMD: −3.64 [95% CI: −6.50 to −0.78], *z* = 2.49, *P* = 0.013, Fig. 5b). In addition, a study reported that the blood plasma NDE Ng in patients with MCI-AD and AD were also significantly lower than those in sMCI patients (*P* < 0.001)^49^.

### Investigation of heterogeneity

To identify the cause of high heterogeneity found in the comparison of CSF Ng levels between AD and healthy control, we performed subgroup analyses, and the results showed that the age-matched and age-mismatched, the MMSE ≥ 20 and MMSE < 20, the Ng ELISA and MSD analytical methods did not affect the heterogeneity (Supplementary Table S6 and Supplementary Figs. S4–S6). But by comparing the types of studies, we found cross-sectional studies have higher heterogeneity (*I*^2^ = 65%, *P* < 0.001, Supplementary Table S6 and Supplementary Fig. S7), while longitudinal studies have the opposite (*I*^2^ = 0, *P* = 0.515, Supplementary Table S6 and Supplementary Fig. S7). And we performed a series of meta-regression analyses and the results showed that age, gender, and MMSE scores could not be regarded as effect factors to explain the heterogeneity between AD and healthy control (*P* > 0.05 for all the analyses).

Similarly, we found the comparison of CSF Ng levels between MCI and healthy control, age matching does not affect heterogeneity (Supplementary Table S6 and Supplementary Fig. S8), and cross-sectional studies have higher heterogeneity (*I*^2^ = 60.5%, *P* = 0.005, Supplementary Table S6 and Supplementary Fig. S9), but longitudinal studies lower (*I*^2^ = 33.8%, *P* = 0.196). Then, in the subgroup analysis of analytical methods, we found that the MSD analytical method has no heterogeneity (*I*^2^ = 43.7%, *P* = 0.149, Supplementary Table S6 and Supplementary Fig. S10), and the ELISA analytical method has higher heterogeneity (*I*^2^ = 66.1%, *P* = 0.001, Supplementary Table S6 and Supplementary Fig. S10). Therefore, the type of studies and Ng diagnostic method are both important sources of heterogeneity regarding CSF Ng levels in patients with AD and MCI.

### Publication bias

We used funnel plot to make a preliminary judgment, and then used the Egger’s test to confirm that there were no publication bias for Ng level comparisons between AD and healthy control, MCI and healthy control, AD and MCI (*P* = 0.156, *P* = 0.110, *P* = 0.156, Supplementary Figs. S11–S13).

## Discussion

Recently, the study of Ng levels in the CSF and blood of patients with AD is a hot topic. In this study, we used meta-analysis to identify the CSF and blood Ng levels for the development of diagnosis biomarkers of patients with AD and mild cognitive impairment (MCI). We extracted a total of 4661 individuals from 24 studies for this meta-analysis and obtained two important findings. Firstly, the level of Ng in CSF of patients with AD and MCI increased, simultaneously, there was higher in patients with AD and MCI-AD than in sMCI patients. And CSF Ng level increased with cognitive declines and negatively associated with MMSE scores. Secondly, the concentration of Ng in blood plasma exosomes of patients with AD and MCI decreased, and Ng in blood plasma exosomes of patients with AD and MCI-AD were also lower than those in patients with sMCI. Therefore, these findings reinforce the clinical evidence that CSF and blood exosomes Ng can be used as a cognitive biomarker for AD and MCI.

For traditional biomarkers, Aβ and Tau are used as diagnostic markers for AD and MCI^53,54^. The former is caused by incorrect cleavage of the amyloid precursor protein, resulting in the aggregation of Aβ monomers into misfolded Aβ oligomers, which can inhibit the glutamate reuptake of neurons and lead to excessive activation of neurons, thereby exerting its neurotoxic effect^55^. In addition, Tau is the main neuronal microtubule assembly protein. In the brain of patients with AD, Tau is abnormally hyperphosphorylated and aggregated into paired helical filaments, which appear as which manifest as neurofibrillary tangles (NFTs)^56^. In the past few years, emerging new biomarkers for early diagnosis of AD have been found, among which, the level of Ng in CSF of AD patients is significantly positively correlated with CSF total Tau and phosphorylated tau^27,31,32,41,47,48^, but the reports of the relationship with Aβ is inconsistent. There was a positive correlation with Aβ40^31^ and a negative correlation with Aβ1-42/Aβ1-40^27^, and also reported that there is no correlation between Ng and Aβ42^32,41^. Unlike the pathological mechanism of Aβ and Tau, Ng responds to synaptic loss or synaptic plasticity disorder in the process of AD, and synaptic integration mirrored by the increase of CSF Ng level may occur earlier than the structural degradation represented by Tau pathology^57,58^. There is considered that synaptic dysfunction and degeneration are a central event in AD pathology from the beginning of the early stages of AD, because learning and memory are formed through synaptic plasticity^56,57^. Comparing with extracellular plaques and neurofibrillary tangles, synaptic loss is directly associated with the degree of dementia, especially hippocampal synaptic functional damage, closely related to memory impairments^59,60^. Therefore, Ng as a post-synaptic membrane protein can be a promising tool for early diagnosis of cognitive decline. Ng binding to calmodulin weakened when the synaptic structure is disrupted, affecting the transmission of Ca^2+^ between the synapses and the formation of LTP, thus it leads to early cognitive decline^61^. And more importantly, studies in humans have shown that the expression of Ng was decreased in the frontal and parietal cortices of AD patients, and Ng was significantly associated with the degree of amyloid and Tau pathology^52,62^. Here, we analyzed follow-up data from 360 MCI patients and showed that the level of Ng in CSF of patients with MCI-AD were obviously higher than that of patients with sMCI^31,32,34,40^. In addition, a meta-analysis also showed that CSF Ng levels are higher in AD and MCI compared to health subjects, and higher in AD compared to MCI^63^. They included 16 articles to clarify whether CSF Ng can be a reliable diagnosis for AD and MCI, and we included 24 articles, which is a larger patient size to analyze the CSF Ng for the diagnosis of AD and MCI. Importantly, we did a series of subgroup analysis and regression analysis to confirm the conclusion. Therefore, the CSF Ng may have the potential to reflect synaptic degeneration at the early stage and can be served as a prognostic cognitive biomarker for MCI-AD and AD.

Although CSF biomarkers are highly accurate in the diagnosis of AD and MCI, they are more traumatic to patients. However, using peripheral blood biomarkers for AD diagnosis has the advantages of less trauma, easy sampling, and widespread adoption, but the sensitivity is lower, thus how to find more sensitive biomarkers in peripheral blood has become a key problem to be solved. The blood plasma neuronally derived exosome provides new insights into reflecting the pathogenesis of AD^64^. Interestingly, we found that, contrary to the level of Ng in CSF, the level of Ng in blood plasma neuronally derived exosomes was decreased in patients with AD and MCI-AD, and there was no change in blood plasma Ng of AD patients. Therefore, the concentration of Ng in the blood plasma exosome was the same as that in the brain tissues of AD patients^52,62^, but the level in CSF was the opposite. At present, there is no specific answer to explain this result. There are literatures showing that 39 endogenous Ng peptides have been identified by combining hybrid immunoaffinity and high-resolution mass spectrometry in CSF of patients with AD, and full-length Ng was modified including disulfide bridges or glutathione^62^. Of which Ng48-76 peptide showed the most significant increase in CSF of patients with AD compared to the healthy control subjects, importantly, Ng48-76 is also the major peptide in AD brain tissue, but this peptide was not found in plasma^34^. Therefore, we speculate that the increase of Ng level in CSF and the decrease of Ng in blood plasma exosomes and brain tissues in patients with AD and MCI-AD are related with the decomposition of Ng into many peptides modified by disulfide bridges or glutathione, and releasing into CSF. This leads to abnormal synapses or even death of synapses and the secretion of synapses-derived exosomes are unavailable. In the future, the sensitivity and accuracy of AD and MCI-AD diagnosis may be improved if the Ng specific peptides can be found in plasma exosomes, and calculate its ratios of peptides to the full length of Ng in CSF and blood exosomes. Recent study reported that the levels of Aβ42, T-tau, and P-T181-tau in CSF were consistent with those of blood exosomes Aβ42, T-tau, and P-T181-tau^65^. These indicate that the change trend of Ng and Aβ1-42 in peripheral blood exosomes is opposite. If they can be detected at the same time, it may be more sensitive to the diagnosis of AD and MCI. In addition, Janelidze et al.^31^ showed that the CSF Ng was higher in patients with chronic ischemic vascular dementia (VD) compared with healthy control subjects. Therefore, Ng also may be considered as a prognostic cognitive biomarker for VD.

Taken together, these findings reinforce the clinical evidence that CSF and blood exosomes Ng can be used as a cognitive biomarker for AD and MCI-AD, but it is still difficult to identify the different stages of the disease through the specific range of Ng values, therefore, further studies are needed to define the Ng values for diagnosis of MCI-AD and AD. In addition, with the development of AD diagnostic criteria, the latest research framework focuses on the diagnosis of AD and MCI using biomarkers, including beta amyloid deposition, pathological tau and neurodegeneration, called AT (N) biomarker system^66^, and the system accepts novel biomarkers added to existing frameworks. Therefore, in the future, the integration of traditional and novel biomarkers of blood exosomes and CSF can be used as a more sensitive and accurate biomarkers system for the diagnosis of AD and MCI.

## Limitations

At the beginning of the meta-analysis, we wanted to extract data from all the articles related to the topic, but after careful scrutiny, we had to exclude some studies that did not have the necessary data. And the funnel plot showed that there were many small sample sizes and low-quality studies. Supplementation of the missing study by the trim and fill method shifted the center line of the funnel plot to the right, which may affect the robustness of the results.

In addition, we found a higher heterogeneity in the comparison of AD patients with healthy controls and MCI patients with healthy controls. The high heterogeneity of the meta-analysis is being mainly due to the type of study and Ng diagnostic method. Thus, in the future, we should adopt longitudinal tracking and appropriate analytical method for diagnosis of Ng in AD and MCI-AD patients.

## Supplementary information

Supplementary Table S1

Supplementary Table S2

Supplementary Table S3

Supplementary Table S4

Supplementary Table S5

Supplementary Table S6

Supplementary Fig. S1

Supplementary Fig. S2

Supplementary Fig. S3

Supplementary Fig. S4

Supplementary Fig. S5

Supplementary Fig. S6

Supplementary Fig. S7

Supplementary Fig. S8

Supplementary Fig. S9

Supplementary Fig. S10

Supplementary Fig. S11

Supplementary Fig. S12

Supplementary Fig. S13
