# Supplementary material for: Neurogranin as a cognitive biomarker in cerebrospinal fluid and blood exosomes for Alzheimer’s disease and mild cognitive impairment
Source: Transl Psychiatry. 2020 Apr 29;10:125. doi: 10.1038/s41398-020-0801-2 (PMC7190828; doi:10.1038/s41398-020-0801-2)
Supplement: Supplementary file 1 — Supplementary Table S1 [file 41398_2020_801_MOESM1_ESM.docx]

**Table S1 Characteristics of included studies.**

| **Author， Year** | **Country** | **Study group** | **N** | **Sex (F/M)** | **Age** | **MMSE** | **Type of Study** | **Sample source** | **Analytical method** |
| --- | --- | --- | --- | --- | --- | --- | --- | --- | --- |
| De Vos et al.,  2015 | Belgium | AD | 20 | 11/9 | 77 (71-84) | 19 (15-23) | cross-sectional | CSF/Blood plasma | ELISA |
|  |  | MCI | 20 | 13/7 | 78 (73-82) | 25 (22-26) |  |  |  |
|  |  | HC | 29 | 18/11 | 48 (42-62) |  |  |  |  |
| De Vos et al., 2016 | Belgium | AD | 50 | 27/23 | 75 (68-78) | 18 (12-23) | cross-sectional | CSF | ELISA |
|  |  | MCI | 38 | 23/15 | 73 (69-79) | 25 (23-27) |  |  |  |
|  |  | HC | 20 | 10/10 | 74 (69-76) | 27 (24-30) |  |  |  |
| Goetzl et al.,  2016 | America | AD | 12丨9 | 6/6丨2/7 | 74.4±6.84丨87.8±2.50 | 26.3±3.45丨28.3±0.96 | cross-sectional | Blood plasma NDE | ELISA |
|  |  | HC | 12丨9 | 6/6丨2/7 | 74.4±6.84丨82.2±2.28 | 29.8±0.39丨21.4±1.60 |  |  |  |
| Headley et al.，2018 | America, Canada  (ANDI) | MCI | 193 | F33% | 75±7 | 27±2 | longitudinal | CSF | MSD |
|  |  | HC | 111 | F50% | 75±6 | 29±1 |  |  |  |
| Janelidze et al.，2016 | Sweden | AD | 74 | F68% | 76.4 (7.4) | 19.4 (3.3) | cross-sectional | CSF | ELISA |
|  |  | MCI | 35 | F66% | 75.0 (7.6) | 26.5 (1.7) |  |  |  |
|  |  | HC | 53 | F70% | 75.3 (6.4) | 28.6 (1.8) |  |  |  |
| Kester et al.，2015 | America | AD | 65 | F45%, 29 | 65 [1] | 22 [0.7] | Longitudinal | CSF | ELISA |
|  |  | MCI | 61 | F38%, 23 | 68 [1] | 27 [0.3] |  |  |  |
|  |  | HC | 37 | F38%, 14 | 64 [2] | 28 [0.3] |  |  |  |

Continued-

| **Author，Year** | **Country** | **Study group** | **N** | **Sex (F/M)** | **Age** | **MMSE** | **Type of Study** | **Sample source** | **Analytical method** |
| --- | --- | --- | --- | --- | --- | --- | --- | --- | --- |
| Kirsebom et al., 2018 | Norwegian | MCI | 20 | F 12 (57%) | 66.8 (7.4) | 26.9 (2.2) | cross-sectional | CSF | ELISA |
|  |  | HC | 20 | F 10 (50%) | 62.8 (9.6) | 29.4 (0.7) |  |  |  |
| Kvartsberg et al., 2015 | Sweden, Netherlands | AD | 100 | 45/55 | 68.6 | 23.2±5.52 | cross-sectional | CSF | ELISA |
|  |  | MCI | 40 | 19/21 | 64.4 | 26.4±2.3 |  |  |  |
|  |  | HC | 80 | 22/58 | 73.6 | 28.6±1.24 |  |  |  |
| Kvartsberg et al.,2015 | Germany | MCI | 25 | 14/11 (56%) | 76 (71 to 78) |  | cross-sectional | CSF/Blood plasma | MSD |
|  |  | HC | 20 | 12/8 (60%) | 54 (41 to 63) |  |  |  |  |
| Lista et al., 2017 | France，Germany，Sweden | AD | 35 | 24/11 | 73 (68-76) | 23 (19-26) | cross-sectional | CSF | ELISA |
|  |  | MCI | 41 | 14/27 | 72 (65-75) | 26 (24-28) |  |  |  |
|  |  | HC | 21 | 13/8 | 64 (59-69) | 30 (29-30) |  |  |  |
|  |  | AD | 95 | 42/53 | 74.6±8 | 24±2 |  |  |  |
| Sun et al.,  2016 | America, Canada  (ANDI) | MCI | 193 | 63/130 | 73.3±7.8 | 27±2 | Longitudinal | CSF | MSD |
|  |  | HC | 111 | 55/56 | 76±5 | 29±1 |  |  |  |

Continued-

| **Author，Year** | **Country** | **Study group** | **N** | **Sex(F/M)** | **Age** | **MMSE** | **Type of Study** | **Sample source** | **Analytical method** |
| --- | --- | --- | --- | --- | --- | --- | --- | --- | --- |
| Pereira et al.，2017 | America, Canada  (ANDI) | AD | 65 | M34/31 | 73.7 (7.6) | 23.5 (1.8) | cross-sectional | CSF | ELISA |
|  |  | MCI | 109 | M67/42 | 74.2(6.9) | 26.7 (1.8) |  |  |  |
|  |  | HC | 57 | M27/30 | 74.8 (5.2) | 29.0 (1.0) |  |  |  |
| Portelius et al.，2018 | America  (ADCC) | AD | 397 | 236/161 | 73 [66-77] | 23 [18-26] | cross-sectional | CSF | ELISA |
|  |  | MCI | 114 | 58/56 | 73 [66-78] | 27 [24-28] |  |  |  |
|  |  | HC | 75 | 50/25 | 69 [61-75] | 29 [29-30] |  |  |  |
| Portelius et al., 2015 | America, Canada  (ANDI) | AD | 95 | 42/53 (44%) | 76 (70-80) | 24 (22-25) | Longitudinal | CSF | MSD |
|  |  | MCI | 105 | 37/68 (35%) | 75 (70-80) | 26 (25-28) |  |  |  |
|  |  | HC | 110 | 55/55 (50%) | 76 (72-78) | 29 (29-30) |  |  |  |
| Sanfilippo et al., 2015 | Brazil | AD | 25 | M6/19 (24%) | 76 (67-85) | 23 (16.5-26) | cross-sectional | CSF | ELISA |
|  |  | MCI | 36 | M14/22 (39%) | 73 (71-76) | 26.5 (24.2-27) |  |  |  |
|  |  | HC | 44 | M13/31 (29.5%) | 71 (67.5-75) | 29 (27-29) |  |  |  |
| Merluzzi et al.，2018 | America  (WRAP, ADRC) | AD | 40 | F35% | 78.3 (6.0) |  | cross-sectional | CSF | ELISA |
|  |  | HC | 25 | F64% | 76.0 (6.5) |  |  |  |  |

Continued-

| **Author，Year** | **Country** | **Study group** | **N** | **Sex (F/M)** | **Age** | **MMSE** | **Type of Study** | **Sample source** | **Analytical method** |
| --- | --- | --- | --- | --- | --- | --- | --- | --- | --- |
| Sutphen et al., 2018 | America, Canada  (ANDI) | AD | 17 | 11/6 (65%) | 74 (6.7) | 23.7 (1.7) | Longitudinal | CSF | ELISA |
|  |  | MCI | 58 | 18/40 (31%) | 74 (6.5) | 26.8 (1.8) |  |  |  |
|  |  | HC | 35 | 3/32 (9%) | 76 (5.7) | 29.1 (1.1) |  |  |  |
| Tarawneh et al.，2017 | America | AD | 95 | 52/43 (55%) | 75.0 (0.8) | 25.3 (0.40) | Longitudinal | CSF | ELISA |
|  |  | HC | 207 | 125/82 (60%) | 72.3 (0.5) | 28.9 (0.09) |  |  |  |
| Vogt et al.，  2018 | America  (WRAP, ADRC) | AD | 40 | F40% (16/40) | 71.9 ± 8.6 | 23.5 (1.8) | cross-sectional | CSF | MSD |
|  |  | MCI | 35 | F31% (11/35) | 73.2 ± 8.5 | 26.7 (1.8) |  |  |  |
|  |  | HC | 335 | F69% (231/335) | 61.9 ± 7.9 | 29.0 (1.0) |  |  |  |
| Wang et al.，  2018 | America, Canada  (ANDI) | AD | 81 | 37 (45.7%) | 74.6 (7.8) | 17.7(13.8-21.3) | cross-sectional | CSF | MSD |
|  |  | MCI | 171 | 58 (33.9%) | 74.2 (7.6) | 27 (25-29) |  |  |  |
|  |  | HC | 99 | 49 (49.5%) | 75.5 (5.3) | 29 (29-30) |  |  |  |
| Wellington et al., 2016 | UK | AD | 100 | 59 (59%) | 63 (57-68) | 21(17-25) | cross-sectional | CSF | ELISA |
|  |  | HC | 19 | 11 (58%) | 61 (50-64) | 30 (29-30) |  |  |  |

Continued-

| Author，Year | Country | Study group | N | Sex (F/M) | Age | MMSE | Type of Study | Sample source | Analytical method |
| --- | --- | --- | --- | --- | --- | --- | --- | --- | --- |
| Wellington et al., 2018 | UK | AD | 68 | 43/25 (63.2%) | 63.1 (6.6) [48-80] | 23 (16-26) [2-30] (Missing = 9) | cross-sectional | CSF | ELISA+MSD |
|  |  | HC | 27 | 16/11 (59.3%) | 58.6 (8.5) [43-71] | 30 (29-30) [28-30] (Missing = 2) |  |  |  |
| Winston et al.,  2016 | America | AD | 10 |  |  |  | Longitudinal | CSF/ Blood plasma NDE | ELISA |
|  |  | MCI | 20 | 13/7 | 68.7±7.76 | 28.82±0.33 |  |  |  |
|  |  | MCI-AD | 20 | 11/9 | 75.35±6.82 | 17.67±0.70 |  |  |  |
|  |  | HC | 20 |  |  |  |  |  |  |
| Winston et al.,  2018 | America | MCI | 61 | F61.5% | 70.2±2.3 | 29.1±0.33 | cross-sectional | CSF | ELISA |
|  |  | HC | 76 | F63.6% | 67.8±2.3 | 27.9±0.64 |  |  |  |

**Abbreviations:** AD, Alzheimer’s disease. MCI, mild cognitive impairment. HC, healthy controls. ADCC, Alzheimer’s Disease Core Center. ADNI, Alzheimer’s Disease Neuroimaging Initiative. WRAP, Wisconsin Registry for Alzheimer’s Prevention. ADRC, Wisconsin Alzheimer’s Disease Research Center. MMSE, Mini-Mental State Examination. CSF, cerebrospinal fluid. ELISA, enzyme-linked immunosorbent assay. MSD, Meso Scale Discovery.
