# Supplementary material for: Neurogranin as a cognitive biomarker in cerebrospinal fluid and blood exosomes for Alzheimer’s disease and mild cognitive impairment
Source: Transl Psychiatry. 2020 Apr 29;10:125. doi: 10.1038/s41398-020-0801-2 (PMC7190828; doi:10.1038/s41398-020-0801-2)
Supplement: Supplementary file 2 — Supplementary Table S2 [file 41398_2020_801_MOESM2_ESM.docx]

**Table S2 The Agency for Healthcare Research and Quality (AHRQ) for the quality assessment of case-control studies.**

| **Author, year** | **1** | **2** | **3** | **4** | **5** | **6** | **7** | **8** | **9** | **10** | **11** | **Total** |
| --- | --- | --- | --- | --- | --- | --- | --- | --- | --- | --- | --- | --- |
| De Vos et al.,  2015 | Yes | Yes | Unclear | Yes | No | Yes | Yes | Yes | Yes | Unclear | No | 7 |
| De Vos et al.,  2016 | Yes | Yes | Unclear | Yes | No | Yes | Yes | Yes | Yes | Unclear | No | 7 |
| Goetzl et al.,  2016 | Yes | Yes | Unclear | Unclear | No | Yes | No | Yes | Unclear | Yes | No | 5 |
| Janelidze et al.，2016 | Yes | Yes | Unclear | Yes | No | Yes | No | Unclear | No | No | No | 4 |
| Kirsebom et al.,  2018 | Yes | Yes | Yes | Yes | No | Yes | Yes | Yes | Unclear | No | Yes | 8 |
| Kvartsberg et al., 2015 | Yes | Yes | Unclear | Yes | No | Yes | Yes | Yes | Unclear | No | No | 6 |
| Kvartsberg et al.,  2015 | Yes | Yes | Unclear | Yes | No | Yes | Unclear | Yes | Unclear | Unclear | No | 5 |
| Lista et al.,  2017 | Yes | Yes | Unclear | Yes | No | Yes | Yes | Yes | Yes | Yes | No | 8 |
| Merluzzi et al.，2018 | Yes | Yes | Yes | Yes | No | Yes | Yes | Yes | Unclear | Yes | No | 8 |
| Pereira et al.,  2017 | Yes | Yes | No | Yes | No | Yes | Yes | Yes | Unclear | No | No | 6 |

Continued-

| **Author, year** | **1** | **2** | **3** | **4** | **5** | **6** | **7** | **8** | **9** | **10** | **11** | **Total** |
| --- | --- | --- | --- | --- | --- | --- | --- | --- | --- | --- | --- | --- |
| Portelius et al.，2018 | Yes | Yes | No | Yes | No | Yes | No | Yes | Unclear | No | No | 5 |
| Portelius et al.,  2015 | Yes | Yes | Yes | Yes | No | Yes | Unclear | Unclear | Unclear | No | No | 5 |
| Vogt et al.,  2018 | Yes | Yes | Unclear | Yes | No | Yes | Yes | Yes | Unclear | Unclear | No | 6 |
| Wang et al.,  2018 | Yes | Yes | Unclear | Yes | No | Yes | No | Yes | Unclear | Unclear | No | 5 |
| Wellington et al., 2016 | Yes | Yes | Unclear | Yes | No | Yes | Yes | Yes | Unclear | Yes | No | 7 |
| Sanfilippo et al., 2018 | Yes | Yes | Unclear | Yes | No | Yes | Yes | Yes | Yes | Unclear | No | 7 |
| Winston et al.,  2016 | Yes | Yes | Yes | Unclear | No | Yes | No | Yes | Unclear | Yes | unclear | 6 |
