# Supplementary material for: Neurogranin as a cognitive biomarker in cerebrospinal fluid and blood exosomes for Alzheimer’s disease and mild cognitive impairment
Source: Transl Psychiatry. 2020 Apr 29;10:125. doi: 10.1038/s41398-020-0801-2 (PMC7190828; doi:10.1038/s41398-020-0801-2)
Supplement: Supplementary file 3 — Supplementary Table S3 [file 41398_2020_801_MOESM3_ESM.docx]

**Table S3 The Newcastle-Ottawa Scale (NOS) for the quality assessment of longitudinal studies.**

| **Author, year** | **Selection** | | | | **Comparability** | | **Exposure** | | | | **Total** |
| --- | --- | --- | --- | --- | --- | --- | --- | --- | --- | --- | --- |
|  | **S1** | **S2** | **S3** | **S4** | **C1** | **C2** | **E1a** | **E1b** | **E2** | **E3** |  |
| Headley et al.，2018 | * | * | - | * | * | * | - | - | * | - | 6 |
| Kester et al.，2015 | * | * | - | * | * | * | - | * | - | - | 6 |
| Sanfilippo et al., 2015 | * | * | - | * | * | * | - | * | * | - | 7 |
| Sun et al.,  2016 | - | * | - | - | * | - | - | - | * | - | 3 |
| Sutphen et al., 2018 | * | * | - | * | * | * | - | - | * | - | 6 |
| Tarawneh et al., 2017 | * | * | - | * | * | * | - | - | * | - | 6 |
| Winston et al.,  2018 | * | * | - | * | * | * | - | - | * | - | 6 |
