# Supplementary material for: Neurogranin as a cognitive biomarker in cerebrospinal fluid and blood exosomes for Alzheimer’s disease and mild cognitive impairment
Source: Transl Psychiatry. 2020 Apr 29;10:125. doi: 10.1038/s41398-020-0801-2 (PMC7190828; doi:10.1038/s41398-020-0801-2)
Supplement: Supplementary file 4 — Supplementary Table S4 [file 41398_2020_801_MOESM4_ESM.docx]

**Table S4 Meta-analysis results of CSF neurogranin levels in patients with AD and mild MCI and HC subjects. AD, Alzheimer’s disease.**

| **Group** | **No. of studies** | **No. of subjects** | | **SMD** | **95%CI** | | **P** | **Heterogeneity** | | | **Publication bias** |
| --- | --- | --- | --- | --- | --- | --- | --- | --- | --- | --- | --- |
|  |  |  |  |  |  |  |  | **Q** | **P** | **I^2^** | **P** |
| AD vs. HC  (CSF) | 19 | 1487 | 1404 | 0.843 | 0.702 | 0.984 | 0.000 | 43.95 | 0.001 | 59.0 | 0.156 |
| MCI vs. HC  (CSF) | 15 | 1077 | 1126 | 0.540 | 0.395 | 0.685 | 0.000 | 30.83 | 0.006 | 54.6 | 0.131 |
| AD vs. MCI  (CSF) | 14 | 1159 | 1057 | 0.185 | 0.066 | 0.303 | 0.002 | 20.44 | 0.085 | 36.4 | 0.156 |
| sMCI vs. MCI-AD  (CSF) | 4 | 170 | 190 | -0.71 | -1.16 | -0.25 | 0.002 | 10.45 | 0.015 | 71.3 | 0.411 |
| AD vs. HC  (plasma) | 2 | 45 | 49 | -0.252 | -0.663 | 0.159 | 0.230 | 0.13 | 0.714 | 0.0 |  |
| AD vs. HC  (NDE) | 2 | 22 | 22 | -9.687 | -20.060 | 0.686 | 0.067 | 15.31 | 0.000 | 93.5 |  |
| MCI vs. HC  (NDE) | 2 | 101 | 86 | -3.637 | -6.498 | -0.776 | 0.013 | 28.47 | 0.000 | 96.5 |  |

**Abbreviations:** MCI, mild cognitive impairment. HC, healthy controls. MCI-AD, mild cognitive impairment patients who progressed to Alzheimer’s disease. sMCI, stable mild cognitive impairment. CSF, cerebrospinal fluid. NDE, neuronal derived exosomes. SMD, standard mean difference. CI, confidence interval.
