# Supplementary material for: Neurogranin as a cognitive biomarker in cerebrospinal fluid and blood exosomes for Alzheimer’s disease and mild cognitive impairment
Source: Transl Psychiatry. 2020 Apr 29;10:125. doi: 10.1038/s41398-020-0801-2 (PMC7190828; doi:10.1038/s41398-020-0801-2)
Supplement: Supplementary file 5 — Supplementary Table S5 [file 41398_2020_801_MOESM5_ESM.docx]

**Table S5 Meta-regression analysis regarding CSF neurogranin levels in patients with AD and MCI and HC subjects.**

| **Group** | **Moderators** | **Coef.** | **95%CI** | | **P** |
| --- | --- | --- | --- | --- | --- |
| AD vs HC | Age | 0.001 | -0.039 | 0.042 | 0.952 |
|  | Gender | 0.803 | -0.823 | 2.428 | 0.312 |
|  | MMSE | 0.011 | -0.724 | 0.944 | 0.781 |
| MCI vs HC | Age | 0.026 | -0.026 | 0.078 | 0.302 |
|  | Gender | 0.020 | -1.426 | 1.466 | 0.977 |
|  | MMSE | -0.249 | -0.495 | -0.003 | 0.047 |

**Abbreviations:** AD, Alzheimer’s disease. MCI, mild cognitive impairment. HC, healthy controls. CSF, cerebrospinal fluid. MMSE, Mini-Mental State Examination. CI, confidence interval.
