# Supplementary material for: Neurogranin as a cognitive biomarker in cerebrospinal fluid and blood exosomes for Alzheimer’s disease and mild cognitive impairment
Source: Transl Psychiatry. 2020 Apr 29;10:125. doi: 10.1038/s41398-020-0801-2 (PMC7190828; doi:10.1038/s41398-020-0801-2)
Supplement: Supplementary file 6 — Supplementary Table S6 [file 41398_2020_801_MOESM6_ESM.docx]

**Table S6** **Subgroup meta-analysis regarding cerebrospinal fluid neurogranin levels in patients with AD and MCI and HC subjects.**

| **Group** | **No. of studies** | **No. of subjects** | | **SMD** | **95%CI** | | **Heterogeneity** | | |
| --- | --- | --- | --- | --- | --- | --- | --- | --- | --- |
|  |  |  |  |  |  |  | **Q** | **P** | **I^2^** |
| AD vs HC  Subgroup |  |  |  |  |  |  |  |  |  |
| Age matched | 13 | 1204 | 746 | 0.779 | 0.615 | 0.944 | 29.66 | 0.003 | 59.5% |
| Age mismatched | 6 | 283 | 610 | 1.020 | 0.728 | 1.311 | 12.70 | 0.026 | 60.6% |
| MMSE≥20 | 12 | 1157 | 823 | 0.857 | 0.684 | 1.030 | 28.71 | 0.003 | 61.7% |
| MMSE＜20 | 4 | 225 | 201 | 0.679 | 0.340 | 1.017 | 7.46 | 0.059 | 59.8% |
| ELISA | 14 | 1123 | 1037 | 0.827 | 0.648 | 1.006 | 35.21 | 0.001 | 63.1% |
| MSD | 4 | 296 | 340 | 0.852 | 0.648 | 1.006 | 7.04 | 0.071 | 57.4% |
| ELISA+MSD | 1 | 68 | 27 | 1.080 | 0.608 | 1.552 |  |  |  |
| Cross-sectional | 14 | 1120 | 904 | 0.555 | 0.359 | 0.751 | 25.34 | 0.005 | 60.5% |
| Longitudinal | 5 | 367 | 500 | 0.499 | 0.335 | 0.662 | 6.04 | 0.196 | 33.8% |

Continued-

| **Group** | **No. of studies** | **No. of subjects** | | **SMD** | **95%CI** | | **Heterogeneity** | | |
| --- | --- | --- | --- | --- | --- | --- | --- | --- | --- |
|  |  |  |  |  |  |  | **Q** | **P** | **I^2^** |
| MCI vs HC  Subgroup |  |  |  |  |  |  |  |  |  |
| Age matched | 10 | 900 | 684 | 0.552 | 0.389 | 0.715 | 20.44 | 0.015 | 56.0% |
| Age mismatched | 5 | 177 | 442 | 0.504 | 0.152 | 0.856 | 10.31 | 0.036 | 61.2% |
| ELISA | 12 | 608 | 806 | 0.532 | 0.340 | 0.723 | 26.11 | 0.006 | 57.9% |
| MSD | 3 | 469 | 320 | 0.568 | 0.349 | 0.786 | 4.44 | 0.108 | 55.0% |
| Cross-sectional | 11 | 660 | 833 | 0.555 | 0.359 | 0.751 | 25.34 | 0.005 | 60.5% |
| Longitudinal | 4 | 477 | 293 | 0.522 | 0.304 | 0.741 | 5.48 | 0.140 | 45.2% |

**Abbreviations:** AD, Alzheimer’s disease. MCI, mild cognitive impairment. HC, healthy controls. CSF, cerebrospinal fluid. MMSE, Mini-Mental State Examination. ELISA, enzyme-linked immunosorbent assay. MSD, Meso Scale Discovery. SMD, standard mean difference. CI, confidence interval.
