# Supplementary material for: Neurogranin as a cognitive biomarker in cerebrospinal fluid and blood exosomes for Alzheimer’s disease and mild cognitive impairment
Source: Transl Psychiatry. 2020 Apr 29;10:125. doi: 10.1038/s41398-020-0801-2 (PMC7190828; doi:10.1038/s41398-020-0801-2)
Supplement: Supplementary file 7 — Supplementary Fig. S1 [file 41398_2020_801_MOESM7_ESM.pptx]

## Slide 1
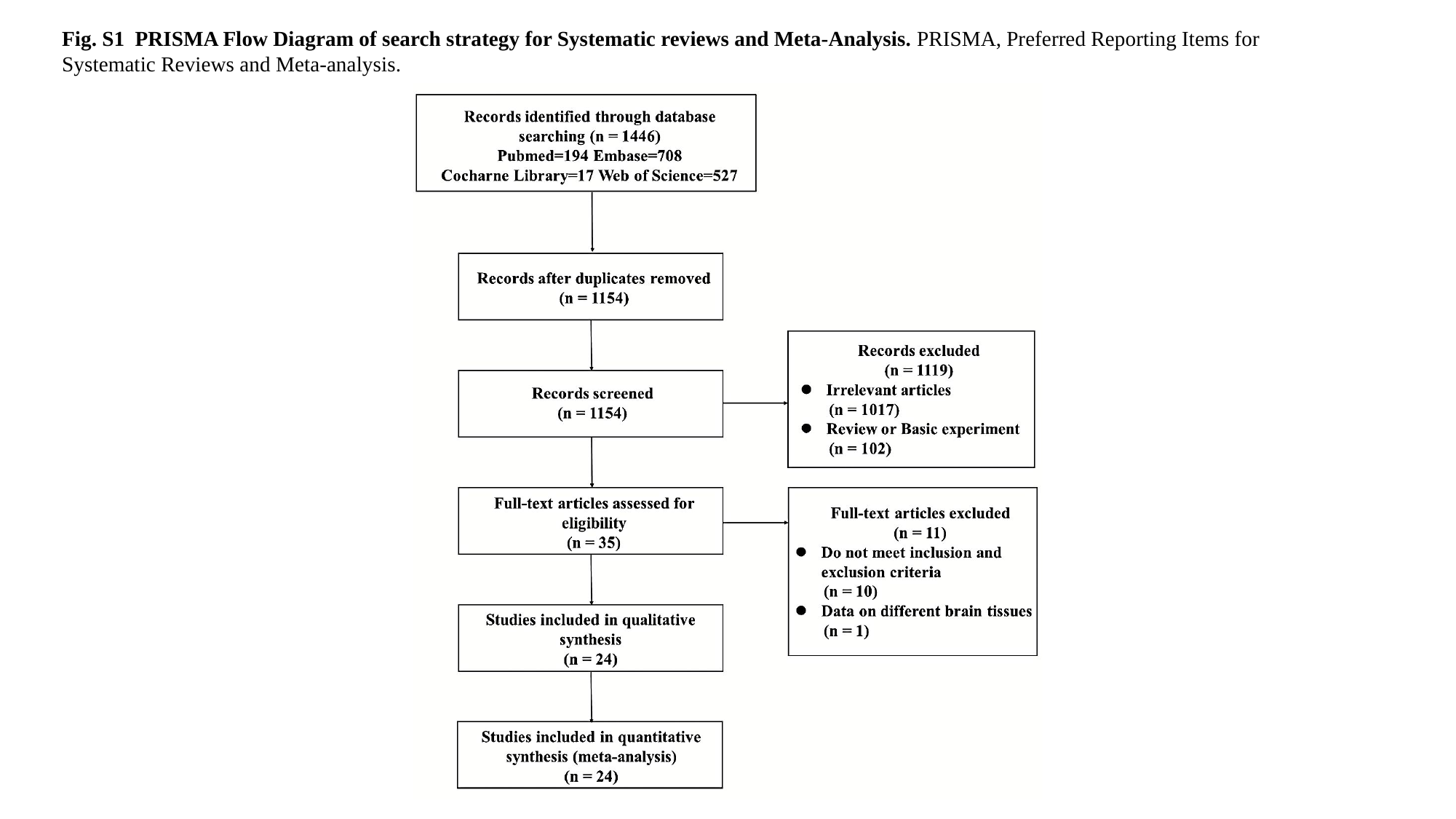

Fig. S1 PRISMA Flow Diagram of search strategy for Systematic reviews and Meta-Analysis. PRISMA, Preferred Reporting Items for Systematic Reviews and Meta-analysis.
