# Supplementary material for: Neurogranin as a cognitive biomarker in cerebrospinal fluid and blood exosomes for Alzheimer’s disease and mild cognitive impairment
Source: Transl Psychiatry. 2020 Apr 29;10:125. doi: 10.1038/s41398-020-0801-2 (PMC7190828; doi:10.1038/s41398-020-0801-2)
Supplement: Supplementary file 8 — Supplementary Fig. S2 [file 41398_2020_801_MOESM8_ESM.pptx]

## Slide 1
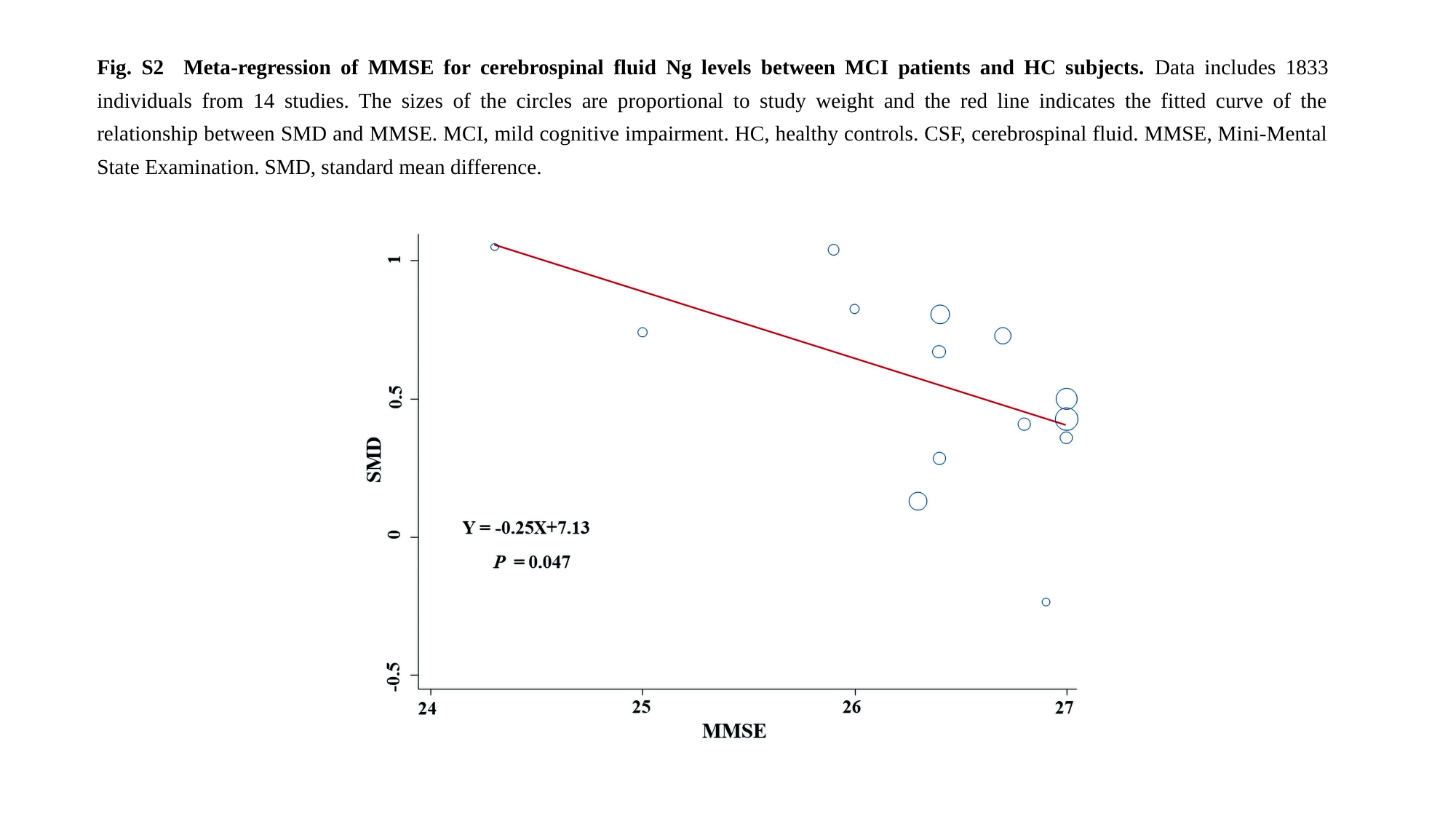

Fig. S2 Meta-regression of MMSE for cerebrospinal fluid Ng levels between MCI patients and HC subjects. Data includes 1833 individuals from 14 studies. The sizes of the circles are proportional to study weight and the red line indicates the fitted curve of the relationship between SMD and MMSE. MCI, mild cognitive impairment. HC, healthy controls. CSF, cerebrospinal fluid. MMSE, Mini-Mental State Examination. SMD, standard mean difference.
