# Supplementary material for: Neurogranin as a cognitive biomarker in cerebrospinal fluid and blood exosomes for Alzheimer’s disease and mild cognitive impairment
Source: Transl Psychiatry. 2020 Apr 29;10:125. doi: 10.1038/s41398-020-0801-2 (PMC7190828; doi:10.1038/s41398-020-0801-2)
Supplement: Supplementary file 9 — Supplementary Fig. S3 [file 41398_2020_801_MOESM9_ESM.pptx]

## Slide 1
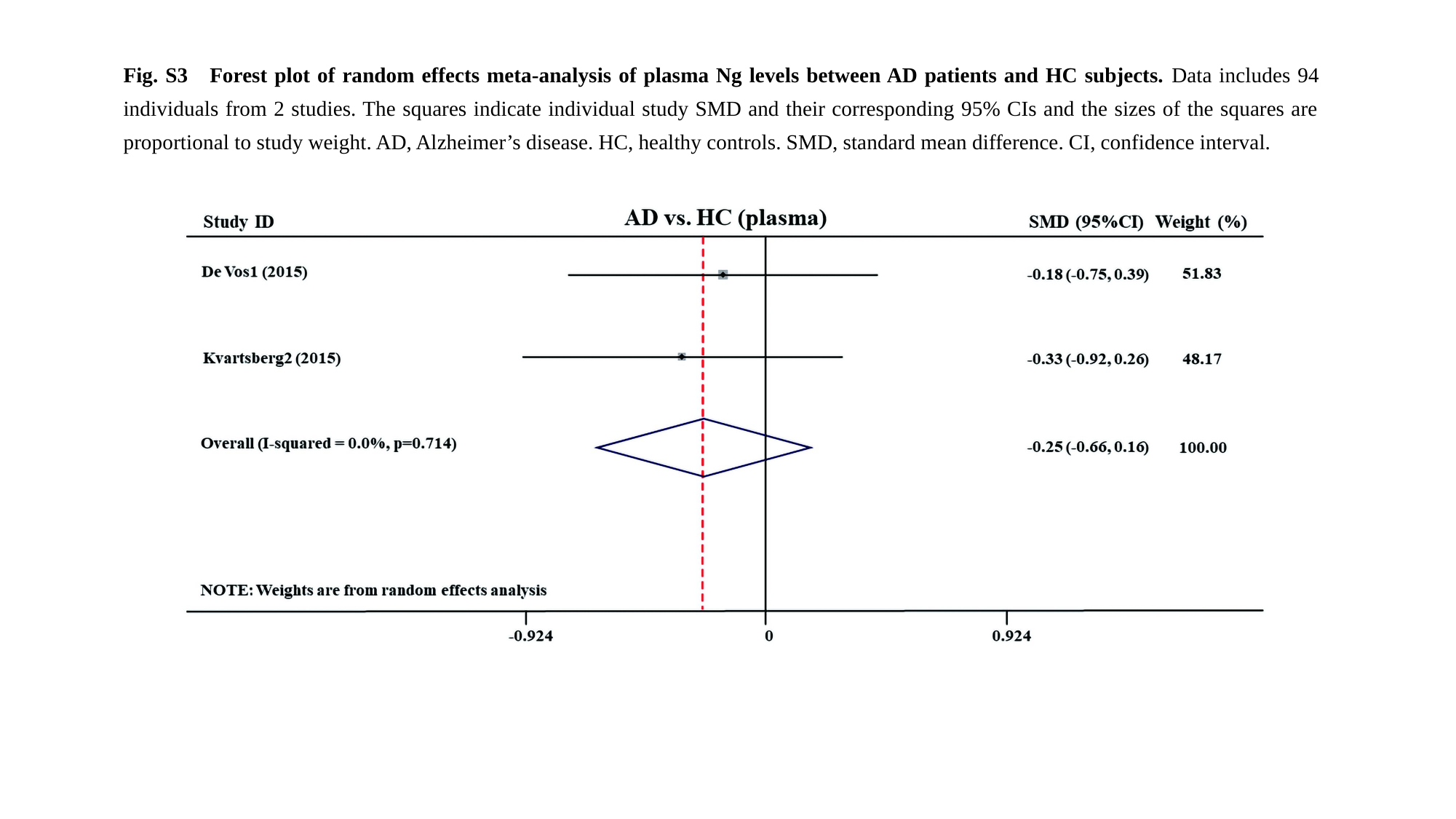

Fig. S3 Forest plot of random effects meta-analysis of plasma Ng levels between AD patients and HC subjects. Data includes 94 individuals from 2 studies. The squares indicate individual study SMD and their corresponding 95% CIs and the sizes of the squares are proportional to study weight. AD, Alzheimer’s disease. HC, healthy controls. SMD, standard mean difference. CI, confidence interval.
