# Supplementary material for: Neurogranin as a cognitive biomarker in cerebrospinal fluid and blood exosomes for Alzheimer’s disease and mild cognitive impairment
Source: Transl Psychiatry. 2020 Apr 29;10:125. doi: 10.1038/s41398-020-0801-2 (PMC7190828; doi:10.1038/s41398-020-0801-2)
Supplement: Supplementary file 11 — Supplementary Fig. S5 [file 41398_2020_801_MOESM11_ESM.pptx]

## Slide 1
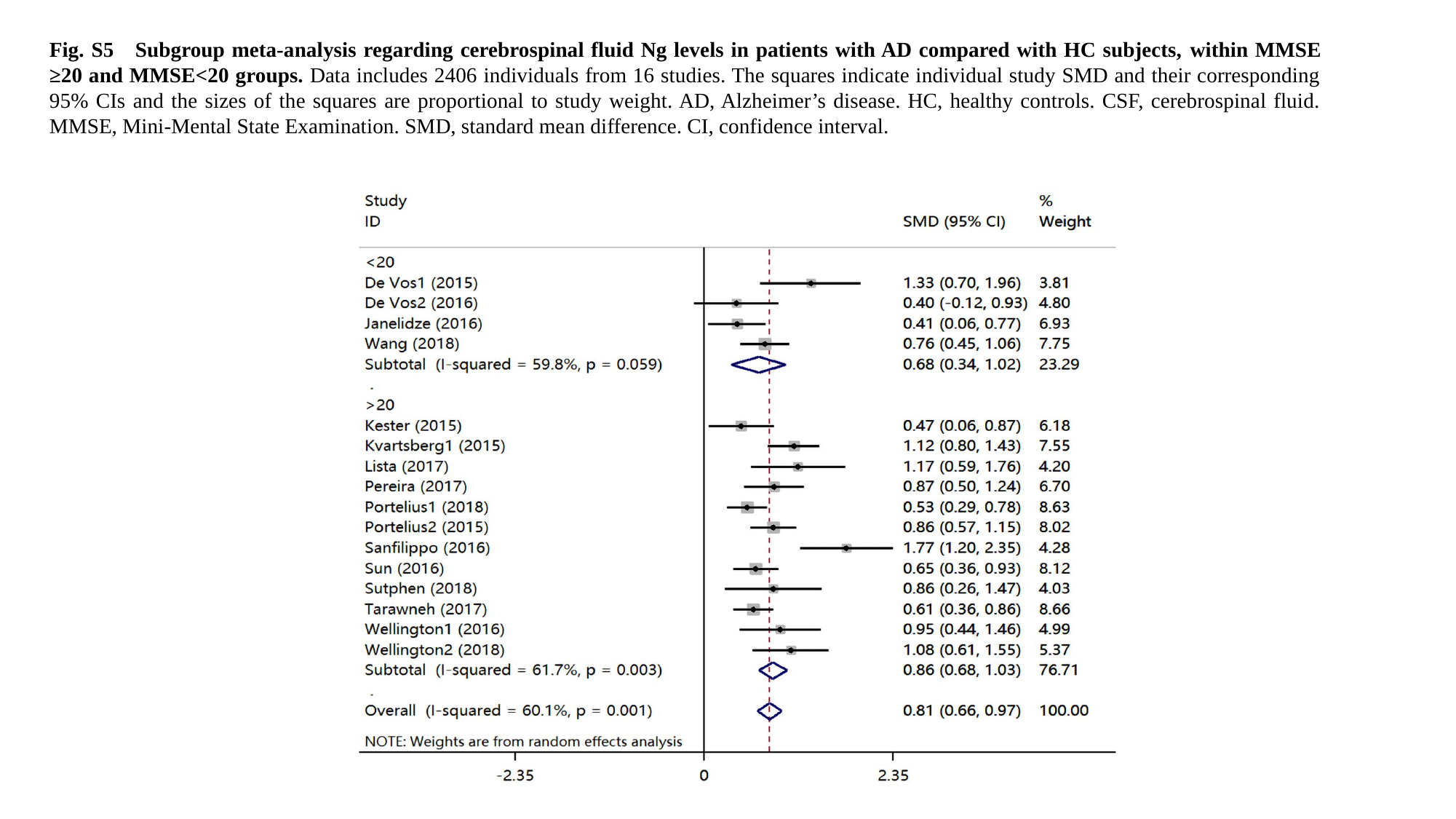

Fig. S5 Subgroup meta-analysis regarding cerebrospinal fluid Ng levels in patients with AD compared with HC subjects, within MMSE ≥20 and MMSE<20 groups. Data includes 2406 individuals from 16 studies. The squares indicate individual study SMD and their corresponding 95% CIs and the sizes of the squares are proportional to study weight. AD, Alzheimer’s disease. HC, healthy controls. CSF, cerebrospinal fluid. MMSE, Mini-Mental State Examination. SMD, standard mean difference. CI, confidence interval.
