# Supplementary material for: Neurogranin as a cognitive biomarker in cerebrospinal fluid and blood exosomes for Alzheimer’s disease and mild cognitive impairment
Source: Transl Psychiatry. 2020 Apr 29;10:125. doi: 10.1038/s41398-020-0801-2 (PMC7190828; doi:10.1038/s41398-020-0801-2)
Supplement: Supplementary file 12 — Supplementary Fig. S6 [file 41398_2020_801_MOESM12_ESM.pptx]

## Slide 1
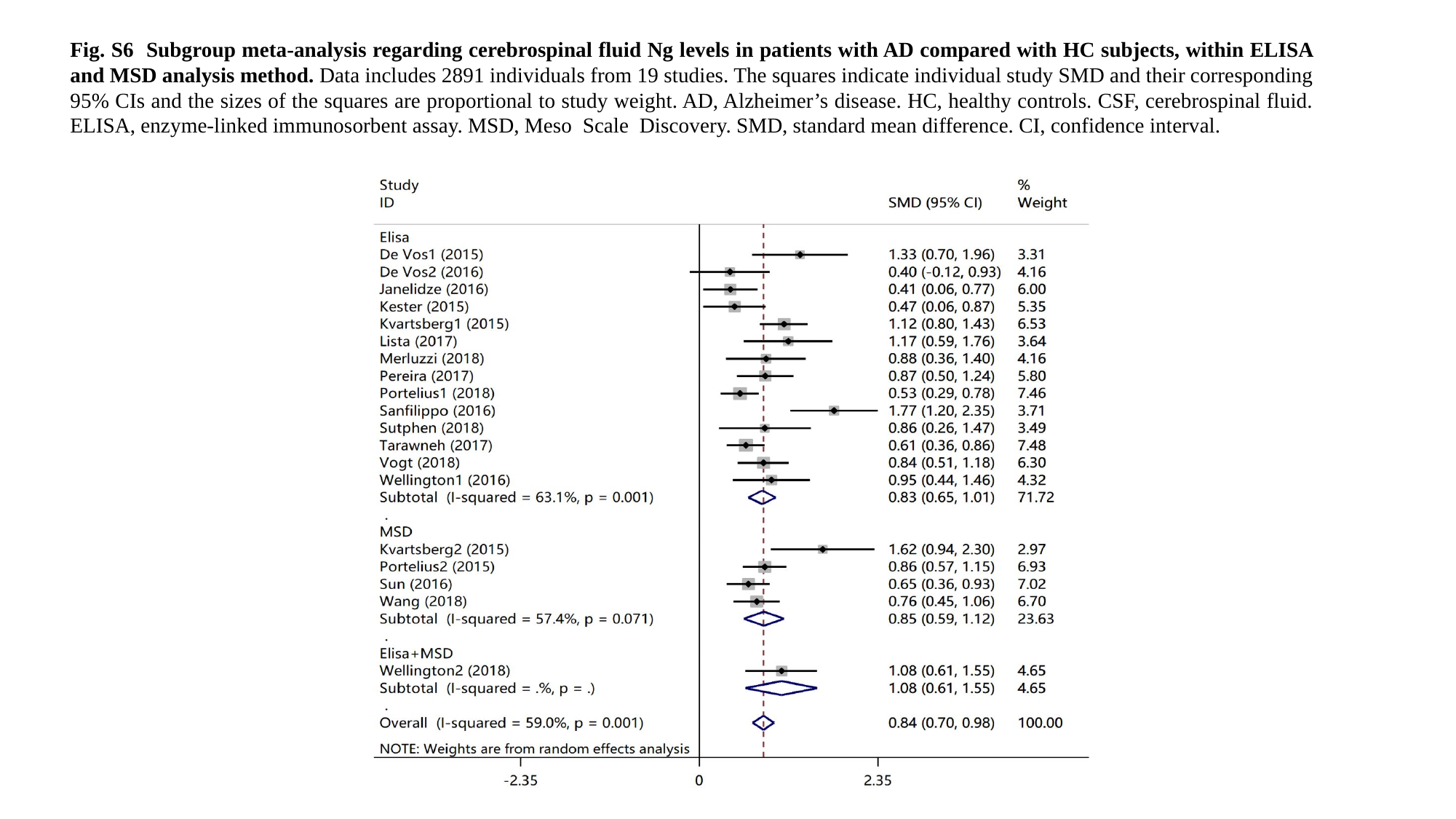

Fig. S6 Subgroup meta-analysis regarding cerebrospinal fluid Ng levels in patients with AD compared with HC subjects, within ELISA and MSD analysis method. Data includes 2891 individuals from 19 studies. The squares indicate individual study SMD and their corresponding 95% CIs and the sizes of the squares are proportional to study weight. AD, Alzheimer’s disease. HC, healthy controls. CSF, cerebrospinal fluid. ELISA, enzyme-linked immunosorbent assay. MSD, Meso Scale Discovery. SMD, standard mean difference. CI, confidence interval.
