# Supplementary material for: Neurogranin as a cognitive biomarker in cerebrospinal fluid and blood exosomes for Alzheimer’s disease and mild cognitive impairment
Source: Transl Psychiatry. 2020 Apr 29;10:125. doi: 10.1038/s41398-020-0801-2 (PMC7190828; doi:10.1038/s41398-020-0801-2)
Supplement: Supplementary file 15 — Supplementary Fig. S9 [file 41398_2020_801_MOESM15_ESM.pptx]

## Slide 1
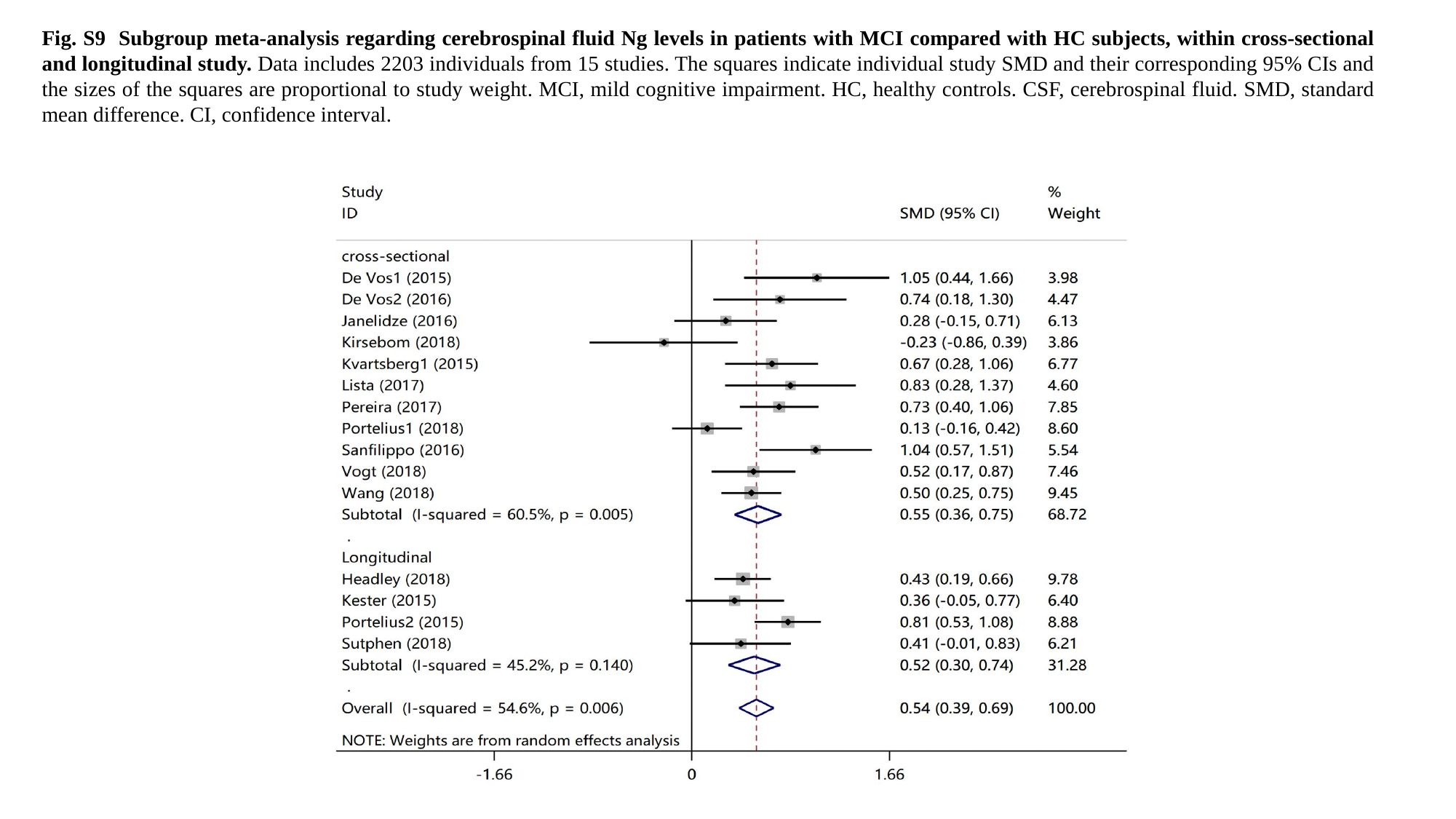

Fig. S9 Subgroup meta-analysis regarding cerebrospinal fluid Ng levels in patients with MCI compared with HC subjects, within cross-sectional and longitudinal study. Data includes 2203 individuals from 15 studies. The squares indicate individual study SMD and their corresponding 95% CIs and the sizes of the squares are proportional to study weight. MCI, mild cognitive impairment. HC, healthy controls. CSF, cerebrospinal fluid. SMD, standard mean difference. CI, confidence interval.
