# Supplementary material for: Neurogranin as a cognitive biomarker in cerebrospinal fluid and blood exosomes for Alzheimer’s disease and mild cognitive impairment
Source: Transl Psychiatry. 2020 Apr 29;10:125. doi: 10.1038/s41398-020-0801-2 (PMC7190828; doi:10.1038/s41398-020-0801-2)
Supplement: Supplementary file 16 — Supplementary Fig. S10 [file 41398_2020_801_MOESM16_ESM.pptx]

## Slide 1
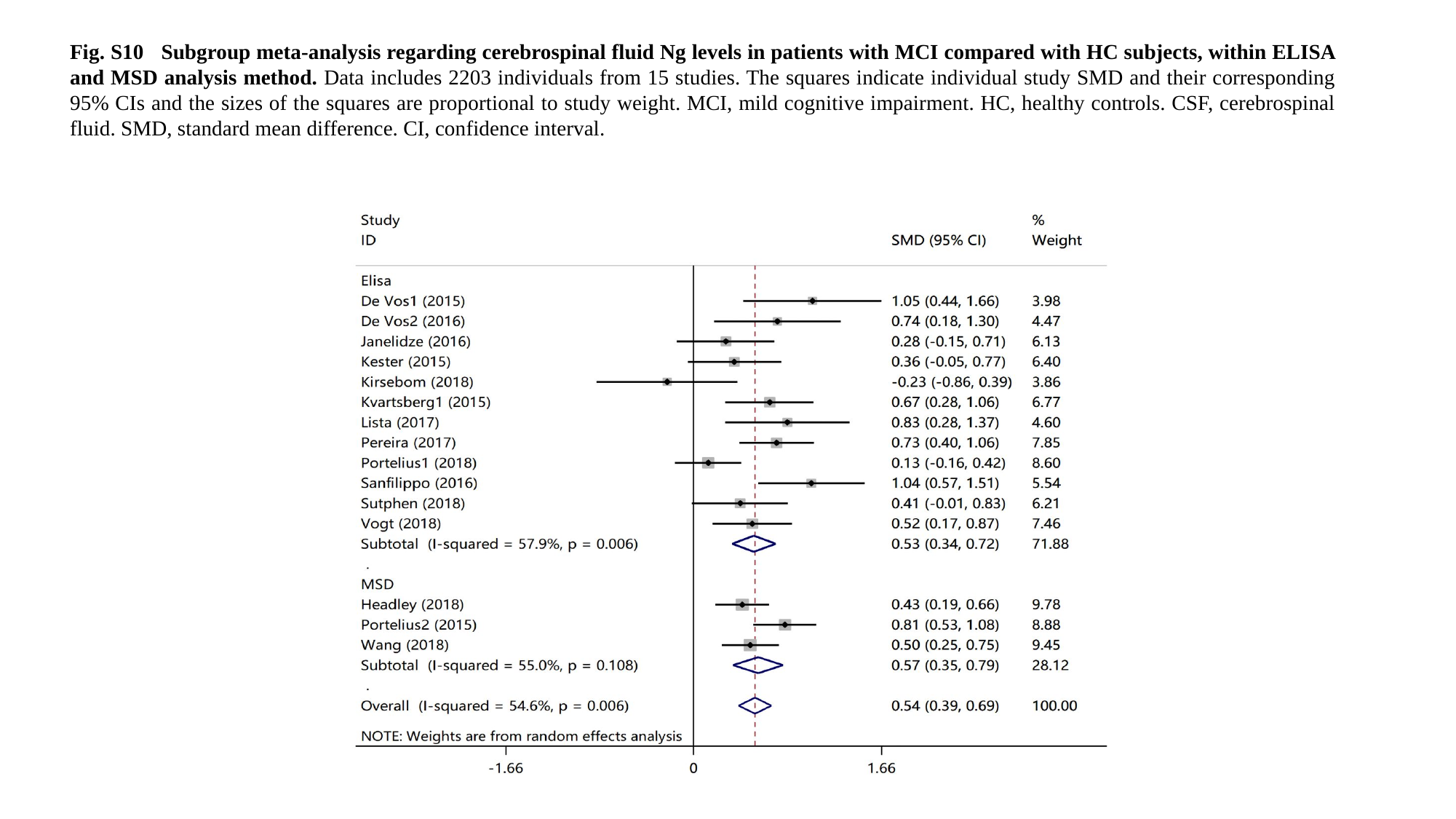

Fig. S10 Subgroup meta-analysis regarding cerebrospinal fluid Ng levels in patients with MCI compared with HC subjects, within ELISA and MSD analysis method. Data includes 2203 individuals from 15 studies. The squares indicate individual study SMD and their corresponding 95% CIs and the sizes of the squares are proportional to study weight. MCI, mild cognitive impairment. HC, healthy controls. CSF, cerebrospinal fluid. SMD, standard mean difference. CI, confidence interval.
