# Supplementary material for: Neurogranin as a cognitive biomarker in cerebrospinal fluid and blood exosomes for Alzheimer’s disease and mild cognitive impairment
Source: Transl Psychiatry. 2020 Apr 29;10:125. doi: 10.1038/s41398-020-0801-2 (PMC7190828; doi:10.1038/s41398-020-0801-2)
Supplement: Supplementary file 17 — Supplementary Fig. S11 [file 41398_2020_801_MOESM17_ESM.pptx]

## Slide 1
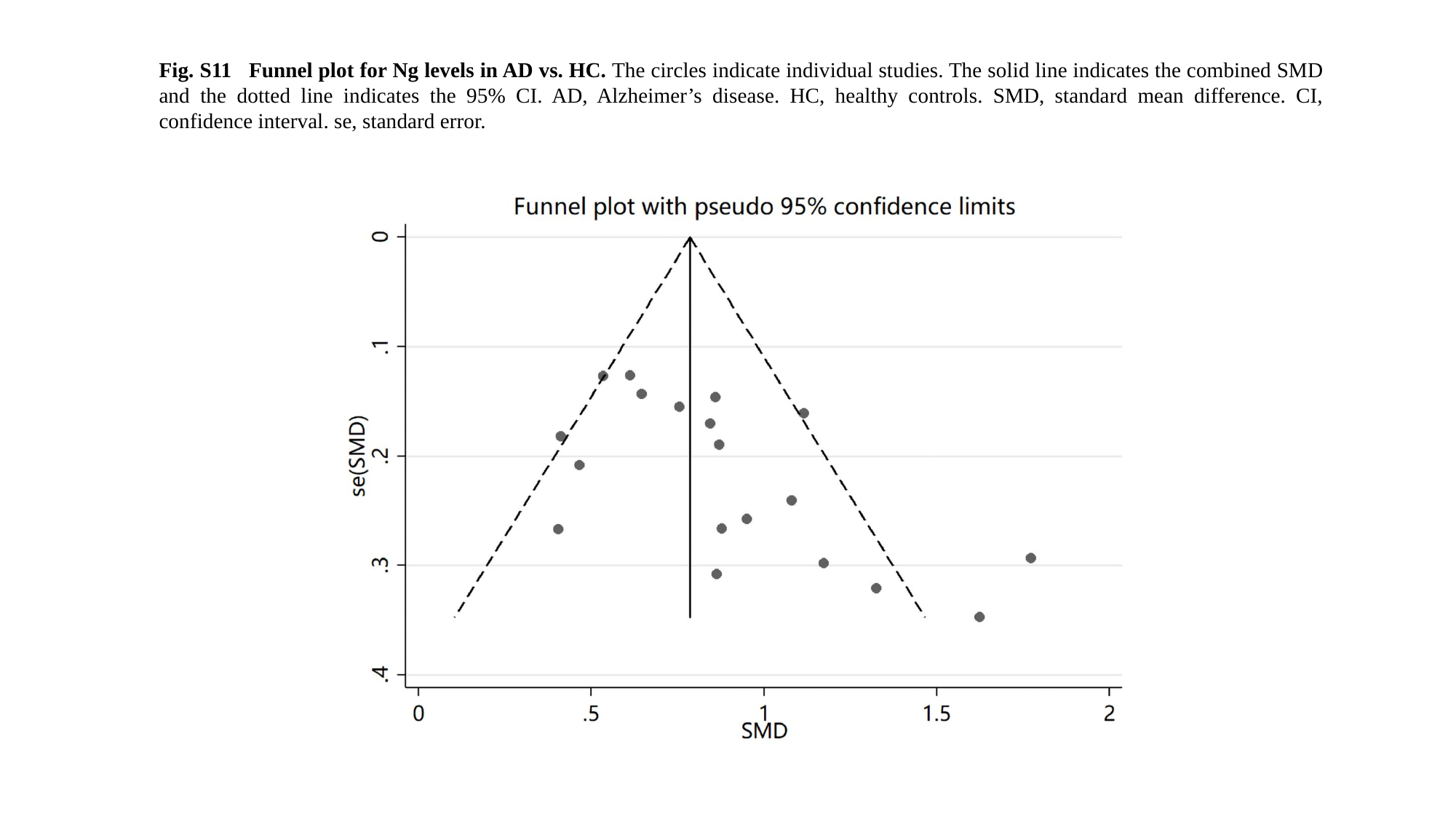

Fig. S11 Funnel plot for Ng levels in AD vs. HC. The circles indicate individual studies. The solid line indicates the combined SMD and the dotted line indicates the 95% CI. AD, Alzheimer’s disease. HC, healthy controls. SMD, standard mean difference. CI, confidence interval. se, standard error.
